# PGC‐1a integrates a metabolism and growth network linked to caloric restriction

**DOI:** 10.1111/acel.12999

**Published:** 2019-07-03

**Authors:** Karl N. Miller, Josef P. Clark, Stephen A. Martin, Porsha R. Howell, Maggie S. Burhans, Spencer A. Haws, Nathan B. Johnson, Timothy W Rhoads, Derek M. Pavelec, Kevin W. Eliceiri, Avtar S. Roopra, James M. Ntambi, John M. Denu, Brian W. Parks, Rozalyn M. Anderson

**Affiliations:** ^1^ Division of Geriatrics, Department of Medicine SMPH, University of Wisconsin Madison Wisconsin USA; ^2^ Department of Biomolecular Chemistry University of Wisconsin Madison Wisconsin USA; ^3^ Biotechnology Center University of Wisconsin Madison Wisconsin USA; ^4^ Laboratory for Optical and Computational Instrumentation University of Wisconsin Madison Wisconsin USA; ^5^ Department of Neuroscience University of Wisconsin Madison Wisconsin USA; ^6^ Department of Biochemistry University of Wisconsin Madison Wisconsin USA; ^7^ Wisconsin Institute for Discovery University of Wisconsin Madison Wisconsin USA; ^8^ Morgridge Institute for Research Madison Wisconsin USA; ^9^ Department of Nutritional Sciences University of Wisconsin Madison Wisconsin USA; ^10^ Geriatric Research, Education, and Clinical Center William S. Middleton Memorial Veterans Hospital Madison Wisconsin USA

**Keywords:** caloric restriction, lipid metabolism, longevity, mitochondria, NAD, PGC‐1a, redox metabolism

## Abstract

Deleterious changes in energy metabolism have been linked to aging and disease vulnerability, while activation of mitochondrial pathways has been linked to delayed aging by caloric restriction (CR). The basis for these associations is poorly understood, and the scope of impact of mitochondrial activation on cellular function has yet to be defined. Here, we show that mitochondrial regulator PGC‐1a is induced by CR in multiple tissues, and at the cellular level, CR‐like activation of PGC‐1a impacts a network that integrates mitochondrial status with metabolism and growth parameters. Transcriptional profiling reveals that diverse functions, including immune pathways, growth, structure, and macromolecule homeostasis, are responsive to PGC‐1a. Mechanistically, these changes in gene expression were linked to chromatin remodeling and RNA processing. Metabolic changes implicated in the transcriptional data were confirmed functionally including shifts in NAD metabolism, lipid metabolism, and membrane lipid composition. Delayed cellular proliferation, altered cytoskeleton, and attenuated growth signaling through post‐transcriptional and post‐translational mechanisms were also identified as outcomes of PGC‐1a‐directed mitochondrial activation. Furthermore, in vivo in tissues from a genetically heterogeneous mouse population, endogenous PGC‐1a expression was correlated with this same metabolism and growth network. These data show that small changes in metabolism have broad consequences that arguably would profoundly alter cell function. We suggest that this PGC‐1a sensitive network may be the basis for the association between mitochondrial function and aging where small deficiencies precipitate loss of function across a spectrum of cellular activities.

## 1 INTRODUCTION

Mitochondrial dysfunction is a prominent feature of aging at the cellular level and includes reduced bioenergetic efficiency and loss of integrity of mitochondrial‐dependent processes linked to cell fate (Sun, Youle, & Finkel, 2016). Numerous studies have reported a decline in expression of nuclear‐encoded genes of the electron transport chain (ETC) with age, and this feature is shared across multiple organisms (McCarroll et al., 2004; Zahn et al., 2006). In mammals, ETC genes are direct or indirect targets of the PGC‐1 (peroxisome proliferator‐activated receptor gamma‐coactivator 1) family of transcription factors. These master regulators of mitochondrial function include PGC‐1a (gene symbol *Ppargc1a*), PGC‐1b (gene symbol *Ppargc1b*), and PRC (PGC‐1‐related coactivator; gene symbol *Pprc1*) (Villena, 2015), although PGC‐1a is by far the best characterized with expression ubiquitous among tissues (Martinez‐Redondo et al., 2016). The pace of aging and incidence of age‐related disease is offset by the dietary intervention of caloric restriction (CR) (Balasubramanian, Howell, & Anderson, 2017). A meta‐analysis study of CR‐induced changes in gene expression identified mitochondrial pathways as the dominant feature in a conserved tissue type‐independent transcriptional signature (Barger et al., 2015), suggesting that PGC‐1a could be a potential target for the development of CR mimetics. Much of the early studies of the biology of PGC‐1a involved relatively high levels of overexpression and focused on exercise or thermogenesis (Scarpulla, 2011). Physiologically, CR interventions in mouse and human studies show modest yet consistent increases in PGC‐1a expression in adipose tissues and liver (Anderson et al., 2008; Corton et al., 2004; Fujii et al., 2017; Nisoli et al., 2005) with less consistent effects reported in skeletal muscle (Gouspillou & Hepple, 2013). Similarly, elevated PGC‐1a expression in adipose and liver in mammalian genetic models of longevity is consistent with enhanced mitochondrial function and efficiency (Bartke & Darcy, 2017). More recently, genetic studies suggest that the actual physiological role of PGC‐1a in adult animals is in adaptation to changes in energy availability or demand rather than maintenance of basal energetics (Villena, 2015). The small but consistent activation of mitochondrial pathways observed with CR is compatible with this newer view of PGC‐1a function. Surprisingly, our knowledge of the function of endogenous PGC‐1a remains quite limited, and the broader consequence of small changes in PGC‐1a status is largely uncharacterized. The goal of this study was to fill in some of these gaps. Specifically, we sought to test whether PGC‐1a activation might be sufficient to mimic CR’s effects, to determine the cellular consequence of modest but persistently augmented PGC‐1a levels, and to identify connections between physiological perturbations of PGC‐1a and metabolism and growth pathways linked to longevity and CR.

## 2 RESULTS

### 2.1 A modest increase in PGC‐1a expression induces large‐scale transcriptional changes

**Figure 1:**
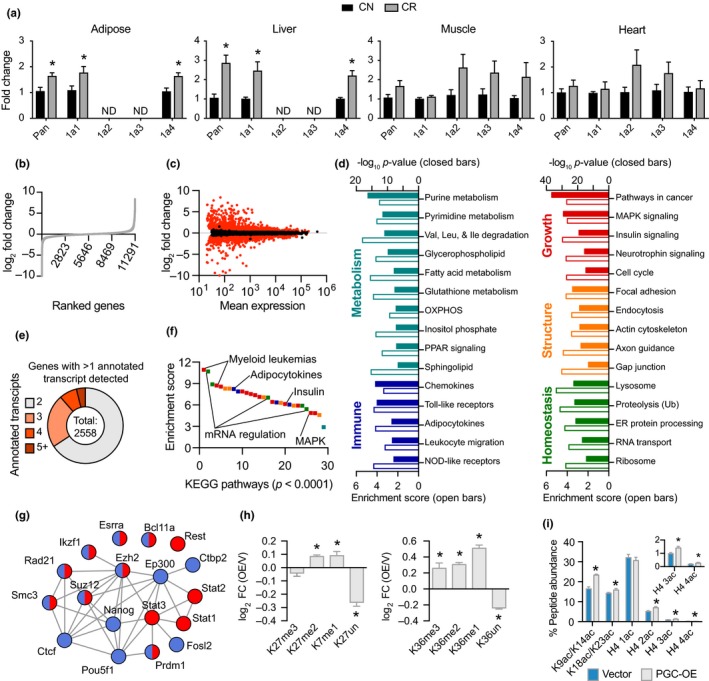
Moderate, stable PGC‐1a overexpression is associated with a large transcriptional network. (a) Detection of PGC‐1a isoform expression in tissues from 12‐month‐old mice on 25% CR from 2 months of age. (b) Ranked fold change of all detected genes between control and PGC‐OE cells and (c) fold change as a function of mean expression with differentially expressed (DE) genes in red (*p* < 0.01, absolute FC > 1.2), *n* = 4. (d) KEGG pathway analysis. (e) Proportion of genes with multiple annotated transcript isoforms. (f) Rank ordered KEGG pathways by enrichment score; colors indicate panel (c) categories. (g) ENCODE factors associated with upregulated (red) and downregulated (blue) DE genes. (h) Fold change of histone H3K27 and K36 methylation and (i) quantitation of histone acetylation by mass spectrometry, *n* = 6. Pan, pan‐PGC‐1a isoform expression; 1a1, PGC‐1a1 isoform, etc. Data shown as means ± *SEM*; asterisk (*) indicates *p* < 0.05 by two‐tailed Student's *t* test

Our previous studies identified growth and metabolic pathways among those most responsive to caloric restriction (Rhoads et al., 2018; Schneider et al., 2017). The induction of mitochondrial oxidative phosphorylation and redox metabolism pathways is a shared response to CR among tissues, suggesting that mitochondrial activation may be at the core of CR’s mechanisms (Barger et al., 2015). Consistent with this, a significant increase in the expression of PGC‐1a transcript was detected in adipose tissue and liver of C3B6‐F1 hybrid mice subject to 25% CR, and although not significantly different in skeletal muscle and heart PGC‐1a transcript levels were numerically higher (Figure 1a). To model the CR program in cultured cells, a stable 3T3‐L1 preadipocyte cell line was generated using viral delivery of PGC‐1a cDNA, hereafter referred to as PGC‐OE cells. Importantly, the ~2‐fold increase in PGC‐1a at the protein level and at the mRNA level compared to control cells paralleled the level of PGC‐1a induction observed in CR tissues (Figure S1A,B).

RNAseq analysis of extracts from PGC‐OE cells identified and quantified transcripts associated with 11,291 unique genes (Figure 1b). Transcripts associated with 5,147 unique genes were differentially expressed in PGC‐OE cells compared to controls (absolute fold change >1.2, BH‐adjusted *p* < 0.01) (Figure 1c; Table S1). PGC‐OE responsive genes represented a range of cellular functions including hormonal regulation, lipid synthesis, intracellular and extracellular remodeling, and nucleotide metabolism. Based on gene ontology terms, 32% of the differentially expressed genes encoded nuclear factors and 50% encoded cytoplasmic factors, including 46% of the MitoCarta (Calvo, Clauser, & Mootha, 2016). Principal component analysis showed clear separation of control and PGC‐OE cells (Figure S1C). At the individual gene level, the top upregulated genes, included cytoskeleton regulatory gene *Dock8*, the tRNA methyltransferase *Trmt61b*, and *Isl1* that binds the enhancer of the insulin gene, expressed exclusively in PGC‐OE (Figure S1D), while the top downregulated genes included *Ror2* a tyrosine kinase receptor implicated in WNT signaling, gap junction protein *Panx1*, and *Igfbp3* IGF binding protein, expressed exclusively in control cells. Interestingly, most of the top responding genes have not previously been linked to PGC‐1a transcriptional co‐activation.

To understand the underlying processes that are responsive to the modest increase in PGC‐1a levels, the differentially expressed genes were categorized by function independent of the directionality of change using KEGG (Kyoto Encyclopedia of Genes and Genomes) via Webgestalt (Zhang, Kirov, & Snoddy, 2005). Pathway analysis revealed 89 pathways enriched in the PGC‐OE cells (BH‐adjusted *p* < 0.0001) (Figure 1d; Table S2). Not surprisingly, PGC‐1a responsive pathways represented a range of mitochondrial processes. Genes in fatty acid oxidation and fatty acid synthesis pathways were differentially expressed, including *Cpt1a* and *Cpt2* in the former, and *Fads1* and *Fads2* and the stearoyl Co‐A desaturases *Scd1* and *Scd2* in the latter (Figure S1E; Table S1). Metabolic processes involving extra‐mitochondrial steps such as amino acid metabolism and nucleotide metabolism were also among those responsive to PGC‐OE. Unexpectedly, immune, growth, structural, and macromolecule homeostatic pathways, including ribosomal and protein processing pathways, were also identified as enriched in the PGC‐OE compared to controls. These data reveal large‐scale changes across multiple cellular processes not limited to mitochondria and not limited to metabolism.

### 2.2 The adaptive response to increased PGC‐1a involves RNA processing and chromatin remodeling

PGC‐OE also impacted processing at the RNA level. More than one annotated transcript isoform was identified for 2,558 genes (Figure 1e). Although the identity of the transcripts for which there were multiple isoforms was the same in PGC‐OE and control cells, there were differences in abundance of transcript isoforms. In the PGC‐OE, 962 of these genes had at least one isoform differentially expressed (*p* < 0.01, absolute fold change >1.2) (Table S2). The top pathways enriched among the transcripts with differential exon usage were growth and RNA processing (Figure 1f). These findings are reminiscent of the hepatic response to short‐term CR in rhesus monkeys, wherein changes in metabolism and growth pathways were associated with the recruitment of RNA processing mechanisms (Rhoads et al., 2018).

The transcriptional response to PGC‐OE involved 46% of total expressed genes identified by RNAseq analysis, substantially more genes than expected. Possible explanations for the scale of the transcriptional response include promiscuous co‐activation of genes that are not ordinarily regulated by PGC‐1a, or alternatively, the transcriptional profile could reflect an adaptive response to chronic PGC‐OE. Published ChIP‐seq experiments identified 1,885 putative PGC‐1a gene targets in HepG2 cells (Charos et al., 2012), which could be considered to be direct targets of PGC‐1a co‐activation. Of those, 738 were identified in this 3T3‐L1 RNAseq dataset, with 334 of those differentially expressed in the PGC‐OE cells, representing only 7% of expressed genes identified. Notwithstanding the difference in cell types between the two studies, these data suggest that the transcriptional response to PGC‐OE extended beyond the known targets of PGC‐1a. To interrogate this further, we tested whether short‐term increases in PGC‐1a gene would recapitulate the transcriptional response detected in the PGC‐OE cells. Two models for acute activation of PGC‐1a were employed, (a) doxycycline‐inducible (DOX) and (b) transient overexpression to increase levels of PGC‐1a, matching the PGC‐1a expression level of the stable line. In each case, transient low level PGC‐1a increased expression of the direct target *Pdk4* within 24 hr, but failed to change expression of metabolic genes activated in the stable PGC‐OE cells and similarly failed to suppress genes involved in growth that were expressed at lower levels in the PGC‐OE cells (Figure S1F,G). These data argue against promiscuous activation of gene expression by PGC‐1a in the stable line.

To investigate factors that could be involved in coordinating an adaptive response to PGC‐OE, we queried the ENCODE database using a gene list with a cutoff of absolute fold change >2 (BH‐adjusted *p* < 0.05) as input (www.encodeproject.org) and visualized using STRING with a medium confidence threshold for protein–protein interactions (Szklarczyk et al., 2015; Figure 1f; Table S3). Among those factors identified were two known PGC‐1a interacting proteins ESRRA and EP300 (Martinez‐Redondo, Pettersson, & Ruas, 2015). Others included chromatin binding and remodeling factors such as RAD21 and SMC3 (Dorsett & Merkenschlager, 2013), the NAD^+^ binding transcriptional corepressor CTBP2 (Chinnadurai, 2007), repressor PRDM1 (Hohenauer & Moore, 2012), and polycomb members EZH2 and SUZ12 (Kashyap et al., 2009). Mass spectrometric analysis of histone modifications (Su & Denu, 2016) provided evidence for chromatin remodeling (Figure S1I), including histone methylation (Figure 1h; Table S4) and acetylation (Figure 1i) that were increased in the PGC‐OE cells. Recent studies suggest close links between metabolism and epigenetic mechanisms of gene expression regulation, in particular due to the importance of histone tail acetylation and methylation in determining chromatin architecture and thereby accessibility (Li, Egervari, Wang, Berger, & Lu, 2018). Altogether these data show a large transcriptional response to PGC‐1a overexpression that appears to be adaptive rather than directly modulated by PGC‐1a and is associated with a network of chromatin‐modulatory factors.

### 2.3 Mitochondrial fuel preference and plasticity are altered with modest PGC‐1a overexpression

**Figure 2:**
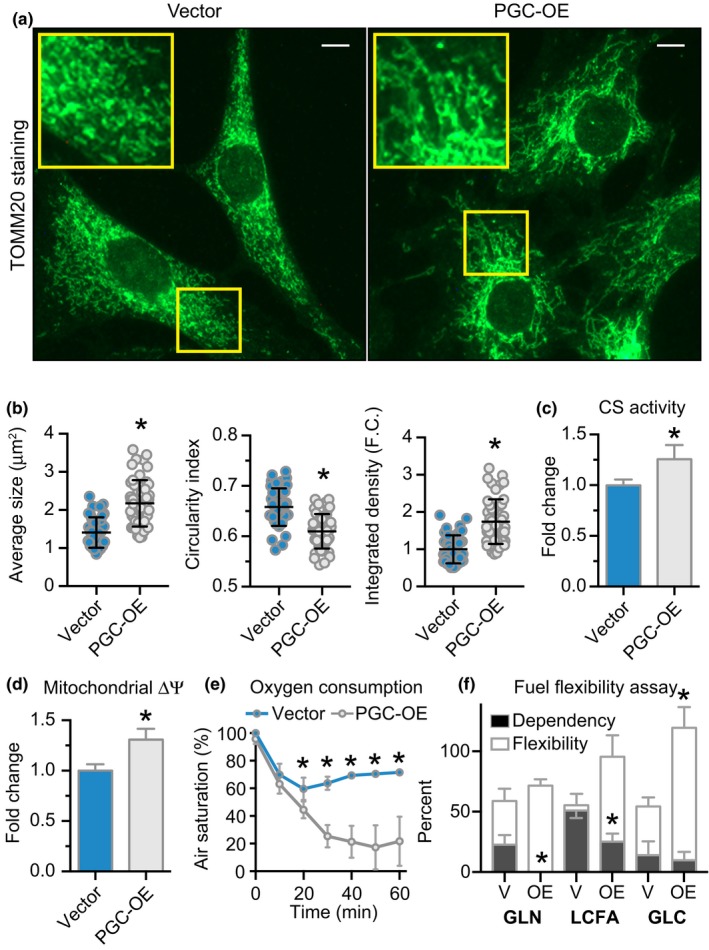
Mitochondrial activation and altered lipid metabolism in PGC‐OE cells. (a) Representative images of TOMM20 immunofluorescence detection (scale bar 10 μm), (b) quantitation of TOMM20 staining by particle size, shape, and integrated density, *n* = 43 vector and 50 PGC‐OE cells. (c) Citrate synthase activity, *n* = 6. (d) JC‐1 w590/w530 measurement of mitochondrial membrane potential, *n* = 15. (e) Oxygen consumption, *n* = 4. (f) Seahorse fuel flexibility assay, *n* = 6. GLN, glutamine; LCFA, long chain fatty acid; GLC, glucose. Data shown as means ± *SD* (b) or means ± *SEM*; asterisk (*) indicates *p* < 0.05 by two‐tailed Student's *t* test

Immunofluorescent detection of the mitochondrial membrane protein TOMM20 revealed differences in mitochondrial network architecture that qualitatively appeared to be more interconnected in PGC‐OE cells (Figure 2a). Quantification of mitochondrial size and circularity indicated that mitochondria were larger and more elongated (Figure 2b). Quantification of stain intensity indicated a modest increase in mitochondrial content (Figure 2b) that was confirmed by a ~1.3‐fold increase in citrate synthase activity (Figure 2c). Mitochondrial membrane potential (ΔΨ_m_) and oxygen consumption were both increased in the PGC‐OE (Figure 2d,e). Consistent with this, significant increases were detected in protein levels of known targets of PGC‐1a including ETC components Mt‐CO1, UQCRC2, and NDUFB8 (Figure S2A). Surprisingly, at the transcript level expression of genes encoding components of the ETC was not overall increased in the PGC‐OE cells (Figure S2B), suggesting the possibility for differences in transcript recruitment or perhaps post‐transcriptional mechanisms of mitochondrial adaptation. These data suggested that the mitochondria in the PGC‐OE were energetically different from those in the control cells. To test this, we investigated fuel preference in both cell types and the ability of mitochondria to switch between fuels when one or another fuel source use was inhibited. In comparison with controls, PGC‐OE showed a complete lack of glutamine dependence and less dependence than controls on mitochondrial long chain fatty acid (LCFA). Mitochondrial capacity was measured by limiting fuel to one source only. Capacity for LCFA fuel use was numerically higher in the PGC‐OE cells, and capacity for glucose as the sole fuel source was significantly higher in the PGC‐OE (Figure 2f; Figure S2C). The lower dependence on any individual fuel source and increased capacity to use different fuel sources suggests greater overall metabolic flexibility in PGC‐OE cells.

**Figure 3:**
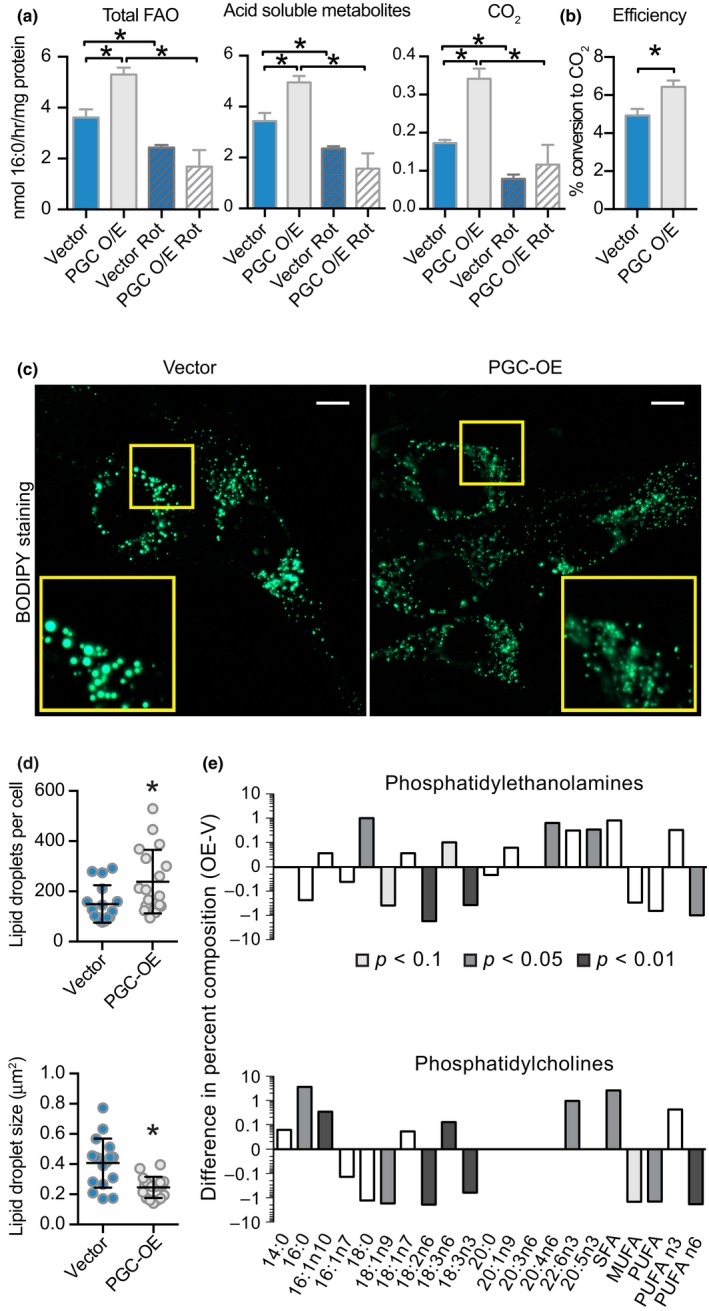
Altered lipid metabolism in PGC‐OE cells. (a) Palmitate fatty acid oxidation radioassay and (b) oxidation efficiency, *n* = 6 or *n* = 3 for rot treatment. (c) BODIPY 493/503 neutral lipid stain representative images (scale bar 10 μm), (d) quantitation of lipid droplet number and average droplet size, *n* = 16 vector and 19 PGC‐OE cells. (e) Phospholipid percent composition represented as the difference between means of PGC‐OE and vector cell lines, *n* = 3. rot, rotenone. Data shown as means ± *SD* (d) or means ± *SEM*; asterisk (*) indicates *p* < 0.05 by two‐tailed Student's *t* test

PGC‐OE cells had an increased basal rate of palmitate (C16:0) oxidation (Figure 3a), consistent with previous studies showing that PGC‐1a regulates fatty acid oxidation (Tabata et al., 2014). Furthermore, the ratio of CO_2_ to palmitate‐derived metabolites indicated that efficiency was enhanced in the PGC‐OE (Figure 3b). Increased fatty acid fuel utilization suggested that there might also be differences in cellular lipid storage in the PGC‐OE cells. Lipid droplets were detected in fixed cells using a fluorescent dye, digitally captured, and quantified (Figure 3c). The PGC‐OE cells had smaller, more numerous lipid droplets than controls (Figure 3d). These data match prior reports of PGC‐1a‐associated changes in lipid storage in cultured muscle cells (Mormeneo et al., 2012) and in skeletal muscle following exercise (Koves et al., 2013). Furthermore, these data are consistent with the Seahorse fuel flexibility assay and the radiolabeled fatty acid oxidation assay that both point to greater lipid turnover in the PGC‐OE cells. To determine the impact of differences in lipid metabolism on lipid membrane composition, total lipid was extracted, distinct lipid classes including phospholipids were isolated by thin layer chromatography, and the fatty acid composition of phosphatidylcholines and phosphatidylethanolamines was determined by gas chromatography. PGC‐OE induced significant differences in phospholipid composition with a marked shift away from saturated fatty acids and toward mono‐ and polyunsaturated fatty acids (Figure 3e; Table S5). Relative levels of essential fatty acids 18:2n‐6 and 18:3n‐3 were higher in phosphatidylcholines and phosphatidylethanolamines from PGC‐OE compared to controls. The composition changes in cellular phospholipids in response to this modest increase in PGC‐1a are reminiscent of the changes in composition of circulating phospholipids induced by CR (Miller et al., 2017).

### 2.4 Cellular redox metabolism is impacted by PGC‐OE

**Figure 4:**
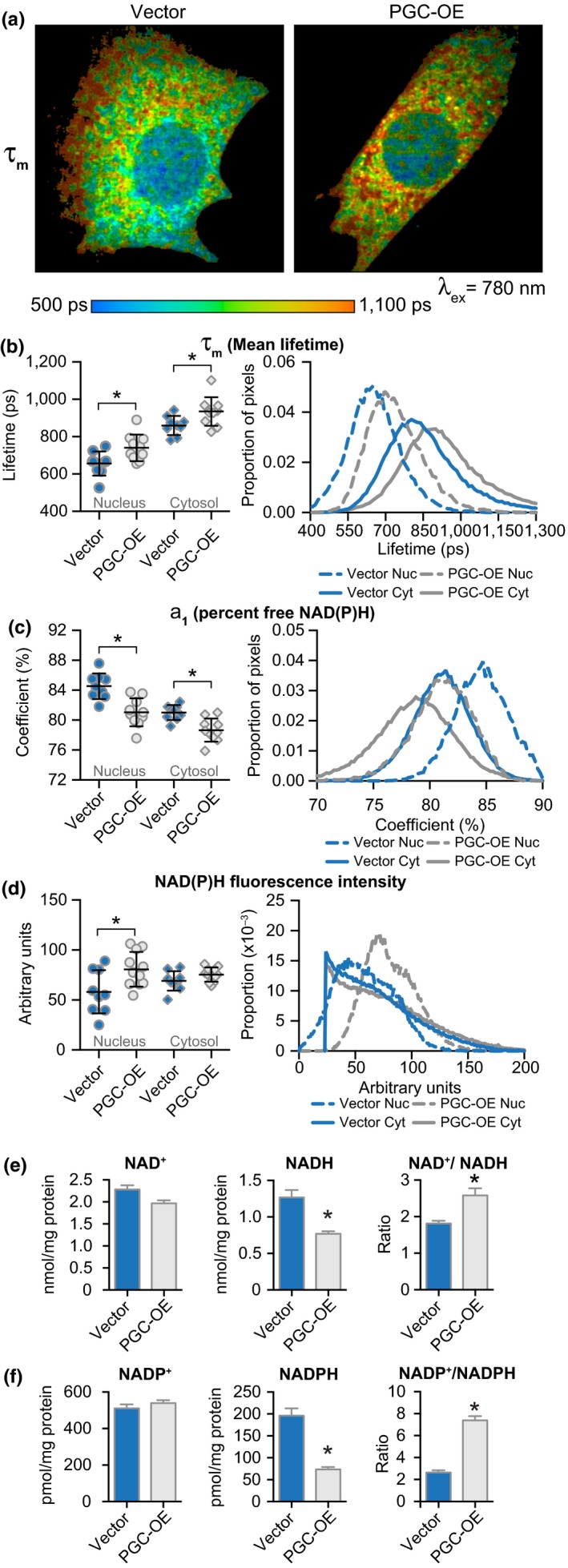
Changes in NAD metabolism associated with PGC‐OE. (a) Representative images of NAD(P)H mean fluorescence lifetime (τ_m_). Quantitation of means (left) and distributions (right) for (b) τ_m_ and (c) a_1_, the proportion of free NAD(P)H, *n* = 9 vector and 10 PGC‐OE cells. (d) Quantitation of NAD(P)H fluorescence intensity, *n* = 9 vector and 11 PGC‐OE cells. (e) NAD and (f) NADP biochemical assays, *n* = 3. Error bars represent ± *SD* or ± *SEM* (e, f). Asterisk (*) indicates *p* < 0.05 by two‐tailed Student's *t* test

Several of the metabolic processes identified above as being linked to PGC‐1a activation are dependent on the redox cofactor nicotinamide adenine dinucleotide (NAD) and its phosphorylated form NADP. Fluorescence lifetime imaging microscopy (FLIM) measures the kinetics of photon release from endogenous pools of NADH and NADPH, informing of the microenvironment of the fluorophores. Fluorescence decay kinetics are characterized by a first‐order decay curve according to the formula: τ_m_ = a_1_•τ_1_ + a_2_•τ_2_, where the fast component (τ_1_) corresponds to free, or unbound NAD(P)H and the slow component (τ_2_) corresponds to protein‐bound NAD(P)H (Miller et al., 2017). The relative contribution of τ_1_ to the decay curve is represented by the coefficient a_1_, effectively a measure of the percent of NAD(P)H in the free state. FLIM data show distinct nuclear and cytosolic cofactor pools with distinct values for τ_m_, and the response to PGC‐OE was subcellular compartment specific (Figure 4a,b). PGC‐OE was associated with an increase in τ_m_ that was explained mostly by a marked decrease in a_1_, the relative contribution of free NAD(P)H (τ_1_) that has faster decay time than bound NAD(P)H (τ_2_) (Figure 4c; Figure S3A,B). Analysis of fluorescence intensity showed a significant increase in nuclear NAD(P)H intensity (Figure 4d). Increased ratio of nuclear:cytosolic NAD(P)H intensity and nuclear:cytosolic τ_m_ in PGC‐OE cells confirmed different responses to increased PGC‐1a within distinct cellular pools (Figure S3C,D). Biochemical assessment of redox ratios in PGC‐OE whole cell extracts (Figure 4e,f) revealed no change in oxidized forms NAD^+^ and NADP^+^ levels, but levels of NADH and NADPH were decreased, leading to an increase in redox ratios in both cases. Because these assays remove protein from cell extracts before analysis, they represent freely available NAD and NADP. Therefore, decreases in levels of free NAD(P)H in these biochemical assays match the decrease in free NAD(P)H as measured by decreased a_1_ in the PGC‐OE. These data suggest a change in redox state in PGC‐OE and furthermore point to cell compartment‐specific responses in NAD(P)H metabolism.

### 2.5 PGC‐OE negatively impacts growth through post‐transcriptional and post‐translational mechanisms

**Figure 5:**
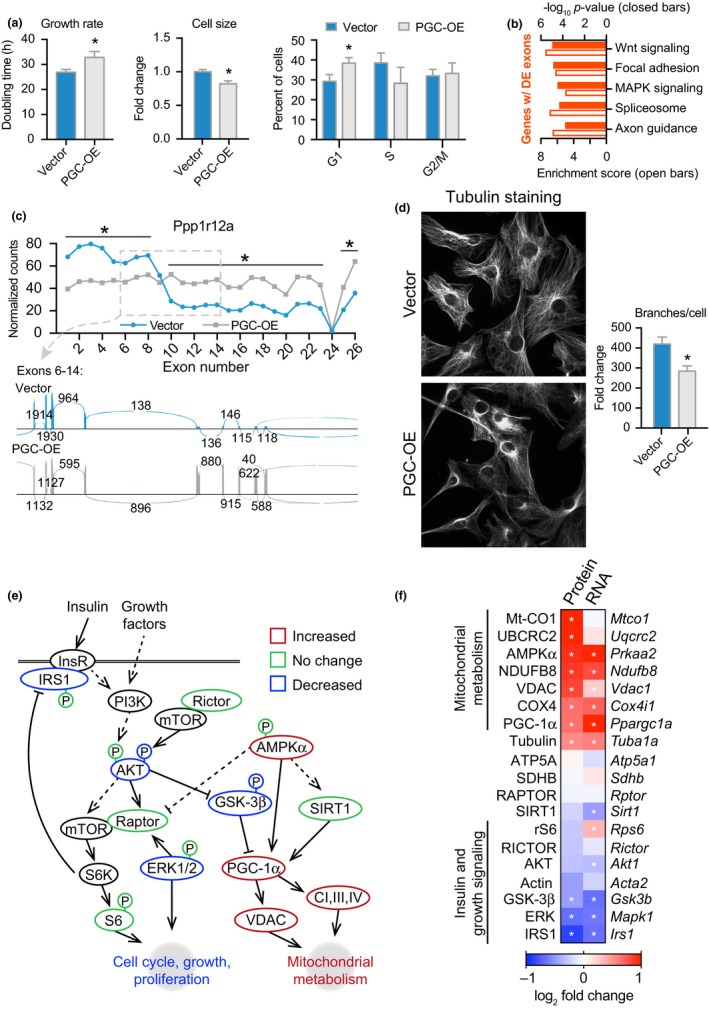
Growth and structural phenotypes of PGC‐OE. (a) Doubling time, *n* = 19, cell size, *n* = 3, and cell cycle phase, *n* = 3. (b) KEGG pathway analysis of genes with ≥1 differentially expressed exon. (c) Exon expression of Ppp1r12a and sashimi plot. (d) Representative images of tubulin immunofluorescent detection and quantitation of tubulin cytoskeletal network branching points, *n* = 39 vector and 43 PGC‐OE cells. (e) Schematic of protein expression and phosphorylation in PGC‐OE by Western blot, *n* = 3. (f) Protein and corresponding RNA levels by RNAseq with the exception of *Ppargc1a* expression by qRT–PCR (see Figure S1E). Data are shown as means ± *SEM*. Asterisk (*) indicates *p* < 0.05 by two‐tailed Student's *t* test or differential expression of exons in (c) and transcripts in (f)

Several cellular growth indices were quantified and showed significantly longer time doubling time, smaller cell size, and delay in cell cycle in the PGC‐OE, where a greater proportion of cells were detected in the G1 phase (Figure 5a; Figure S4A). The enrichment of the spliceosome and RNA transport pathways raised the possibility that gene expression regulation could be exerted through changes in exon usage independent of differences in total abundance. DEXSeq exon counting analysis of the PGC‐OE transcriptome revealed a subset of genes with significant differences in exon usage including 1,033 differentially counted exons representing 635 genes. Growth and structural pathways were predominant in the KEGG pathway analysis (Figure 5b; Figure S4B; Tables [Link], [Link] and [Link], [Link]), suggesting that growth regulation in particular was responsive to PGC‐1a status through RNA‐based mechanisms. The majority of transcripts with differential exon usage in the PGC‐OE cells involved differences in one exon only; however, in 159 instances more than one exon was differentially incorporated. Examples included *Ppp1r12a*, a myosin phosphatase regulatory subunit involved in cell division and migration in multiple cell types (Matsumura & Hartshorne, 2008; Figure 5c) and *Mtor*, a central regulator of growth signaling (Kennedy & Lamming, 2016; Figure S4C). Consistent with the gene expression profiling data, changes in the cytoskeleton were evident. Overt changes in tubulin cytoskeletal morphology were revealed by immunodetection, including prominent perinuclear accumulation and reorganization of tubulin networks in cytosolic regions (Figure 5d). The structural changes may be linked to differences in mitochondrial distribution and morphology described earlier. In contrast, no obvious change in actin cytoskeletal morphology was detected (Figure S4D). Growth signaling pathways were also downregulated in PGC‐OE. Abundance and modification status of factors involved in insulin, mTOR, and WNT signaling were significantly different in the PGC‐OE (Figure 5e; Figure S4E). AKT and GSK‐3β (glycogen synthase kinase 3‐beta) were decreased, and AMPKα (AMP‐activated protein kinase alpha) levels were increased in PGC‐OE cells compared to control cells. mTOR complex 1 receives additional inputs from insulin signaling which is partially mediated by IRS1 (Yoon, 2017). IRS1 protein levels were substantially lower in PGC‐OE cells. Although a change in AKT T308 phosphorylation downstream of IRS1 was not detected, activating phosphorylation at S473 (mTOR complex 2 target) was lower in PGC‐OE cells and phosphorylation of AKT target GSK‐3β at S9 was also lower, suggesting a decrease in AKT activity. Levels of the growth‐associated kinase ERK (extracellular signal‐regulated kinase) were also lower in PGC‐OE. Changes in growth regulatory parameters at the protein level were highly coordinated with changes at the transcript level (Figure 5f). These data potentially place growth parameters downstream of the metabolic changes induced by a modest increase in PGC‐1a levels.

### 2.6 PGC‐1a is linked to growth and metabolic networks in vivo

**Figure 6:**
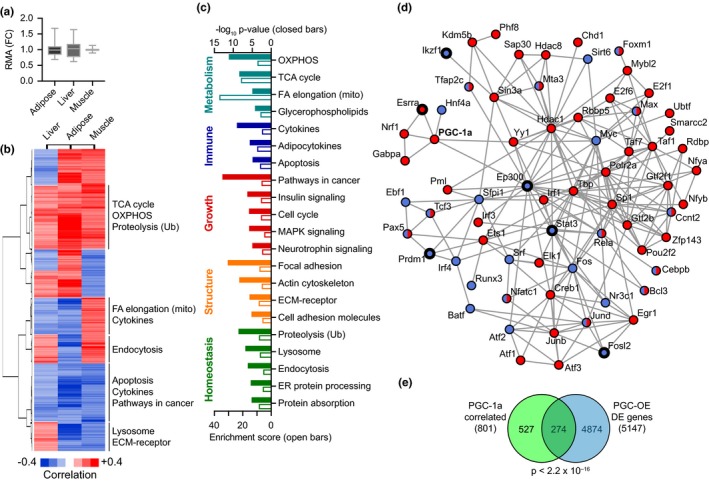
Tissue type‐independent PGC‐1a core gene network. (a) Range of PGC‐1a transcript expression in a diverse panel of hybrid mouse lines relative to mean expression (boxes indicate second and third quartile; whiskers indicate minimum and maximum). (b) Hierarchical clustering and cluster‐specific KEGG pathways for 801 genes correlated with PGC‐1a expression independent of tissue type and (c) KEGG pathways for entire set of 801 genes. (d) ENCODE analysis of factors associated with expression of genes positively (red) or negatively (blue) correlated with PGC‐1a expression independent of tissue type. Factors common to Figure 1h in thick outlines. (e) Overlap of gene identity between PGC‐1a‐correlated genes and PGC‐OE differentially expressed genes, *p*‐value calculated by Fisher's exact test.

Previous studies have largely relied on genetic manipulation of PGC‐1a in cell culture models or in specific tissues; however, the suite of factors responsive to differences in endogenous PGC‐1a in vivo has yet to be defined. The Hybrid Mouse Diversity Panel (HMDP) is a genetic reference population composed of more than 100 commercially available mouse strains with gene expression analysis conducted for several tissues (Bennett et al., 2010; Parks et al., 2013, 2015). These published data were used to investigate strain‐dependent differences in expression of endogenous PGC‐1a in liver, epididymal white adipose tissue, and skeletal muscle. The range of abundance of *Ppagrc1a* transcript was remarkably tight among the 100 strains, with coefficients of variance lower than 25% (Figure 6a). To investigate whether there were patterns of gene expression that tracked with innate PGC‐1a status based on abundance, genes with transcripts correlating with PGC‐1a expression were identified (biweight mid‐correlation *p* < 0.05). In addition to tissue‐specific gene correlations (Figure S5), a core group of 801 genes significantly correlated with PGC‐1a expression across all three tissues (Figure 6b; Table S1). The directionality of the correlation was not equivalent among tissues for 60% of the core network, but a subset was consistently correlated with PGC‐1a across tissues. Pathway analysis via KEGG of the 168 genes consistently positively correlated with PGC‐1a expression identified TCA cycle, OXPHOS, and ubiquitin‐mediated proteolysis pathways, while the 155 genes consistently negatively correlated with PGC‐1a expression represented immune, inflammation, and growth pathways. To understand the extent of the PGC‐1a‐linked network, the entire set of core correlated genes were grouped independent of directionality and used for pathway analysis, the same method that was used for the PGC‐OE transcriptome (Figure 1d). A suite of pathways was revealed including metabolic, structural, growth, homeostatic, and immune pathways (Figure 6c; Table S2), presenting a pattern almost identical to that identified in the PGC‐OE preadipocyte line. A gene list of the 323 consistently positively and negatively PGC‐1a correlated genes was used to query the ENCODE database, and a suite of factors was identified including PGC‐1a itself. Using STRING and limiting the output to only factors with high confidence interactions, a highly interconnected network was identified with 40 factors associated with positively correlated genes, 17 factors with negatively correlated genes, and 12 factors with both (Figure 6d; Table S3). A subcluster of known PGC‐1a interacting transcription factors involved in mitochondrial regulation was identified including ESRRA, NRF1, GABPA, and HNF4A. This subcluster was connected to the rest of the regulatory network via PGC‐1a through YY1 and EP300, general transcription regulators and established PGC‐1a‐associated factors. Overlap at the level of gene identity between the core network of PGC‐1a‐correlated genes and the group of differentially expressed genes in the PGC‐OE preadipocyte line was significant by Fisher's exact test (Figure 6e). These data attest to the physiological relevance of the findings of the PGC‐OE experiments and indicate that the breadth of the network identified in culture cells reflects the network of genes linked to PGC‐1a status in vivo*.*

## 3 DISCUSSION

Transcriptional analysis and functional cellular assays indicated that several distinct mechanisms were engaged in integrating metabolic and growth phenotypes. In addition to adaptive regulation at the level of transcript abundance, there was evidence of extensive chromatin remodeling. Global nontargeted analysis revealed significant changes in modification of histone tails by acetylation and methylation. These changes are likely linked to availability of metabolites (Li et al., 2018), and it will be of considerable interest to determine the genomic location of histone marks that respond to changes in mitochondrial function and specific pathways involved. RNA‐based mechanisms were also evident in the PGC‐OE, where changes in RNA processing were identified at the level of transcript isoform abundance in pathways of growth and metabolism. Independent of transcript abundance, changes in exon usage were also specifically engaged for genes in growth‐associated pathways. Interestingly, both altered isoform abundance and differential exon usage in the absence of changes in overall transcript abundance were recently reported to be engaged in the early response to CR in nonhuman primates (Rhoads et al., 2018), where again the target pathways are metabolism and growth regulation. Another completely distinct mechanism involved changes in cell signaling at the protein post‐translational modification level. Growth signaling plays a highly conserved role in longevity regulation, and the investigation of both mTOR and insulin/IGF signaling is a very active area of research (Kennedy & Lamming, 2016; Manning & Toker, 2017; Yoon, 2017). The PGC‐OE cells showed critical differences in signaling through these growth regulatory nodes. We suggest that changes in growth signaling may ultimately underlie differences in immune and inflammatory pathways detected at the transcript level; there is extensive cross talk between signaling factors associated with growth and those downstream of cytokine and innate immune receptors (Osborn & Olefsky, 2012).

The modest induction of PGC‐1a among tissues from CR fed mice was not unexpected. Recent studies of CR across tissues in mice and other species place mitochondrial pathways at the core of a tissue type‐independent CR responsive network (Barger et al., 2015). Further evidence for CR‐induced mitochondrial adaptation comes from independent observations that mice on CR preferentially use lipid as a source of fuel (Bruss, Khambatta, Ruby, Aggarwal, & Hellerstein, 2010) and have significantly altered lipid profiles (Miller et al., 2017). The PGC‐1a‐associated change in cellular lipid metabolism shown here featured differences in chain length and degree of saturation of phospholipids. These membrane‐resident lipids are likely to impact cellular membrane structure and fluidity both within the cell and at the cell surface (Harayama & Riezman, 2018), and their influence may even extend to the function of proteins embedded within cellular lipid layers (Vitrac et al., 2016). The shift in mitochondrial energy metabolism toward increased respiration and enhanced metabolic flexibility was not confined to mitochondrial pathways but extended more broadly to redox metabolism including levels, redox ratios, and chemical properties of NAD(P)H. The detection of discrete nuclear and cytosolic pools of NAD(P)H corroborates recent reports (Cambronne et al., 2016; Ryu et al., 2018), but the fact that they appear to be independently responsive to PGC‐1a status raises new questions about how these pools are established, maintained, and independently regulated. A major prediction of a mitochondrial‐centric model of CR mechanisms is that at least some of the cellular, tissue, and whole‐body effects of CR are contingent on metabolic status, that is, responsive to an imposed change in energetics. Consistent with this, cellular aspects of CR (delayed growth, enhanced respiration, preference for lipid fuel utilization) are mirrored in cells with activation of mitochondria via modest increases in PGC‐1a. These findings are further corroborated in vivo by the identification of the same pathways as innately sensitive to differences in PGC‐1a expression, a surrogate for mitochondrial status.

The genetics of human longevity has been an active area of investigation, and genes associated with longevity include ApoE, FOXO3a, and AdipoQ. A recent meta‐analysis of longevity‐associated quantitative trait loci identified PPARg as a potential longevity factor (Hook et al., 2018). The close relationship between growth inhibition and activation of mitochondrial oxidative pathways could be a more general mechanism for cellular homeostasis. A switch toward respiration is predicted to tip the balance away from growth by diminishing the availability of redox factors and metabolites required for anabolic and anaplerotic pathways. Data from this study prompt a revision of the consensus view that mitochondria simply respond; a new perspective would need to allow for the inverse paradigm where cellular function can be dictated by mitochondrial status. We propose that modest differences in the status of the mitochondria have far‐reaching consequences in terms of cellular metabolism and growth, and that these established longevity pathways might be harnessed through PGC‐1a.

## 4 EXPERIMENTAL PROCEDURES

Full experimental procedures are included in the Supporting Information.

### 4.1 Cell culture, qRT–PCR, Western Blot, and immunofluorescence

Conducted using standard techniques. pcDNA3.1‐PGC1a cDNA (D. Kelly, WUSTL) was subcloned into lentiviral transfer vector pWPXL (Addgene), and 3T3‐L1 cells stably overexpressing PGC‐1a were generated by pWPXL‐PGC‐1a viral delivery. Clonal cell lines were isolated and assessed by PGC‐1a expression, and the same vector clonal line and PGC‐OE clonal line were used for all experiments. Metabolic phenotypes of PGC‐OE were validated in an independent fibroblast cell line (Figure S6). Cells used in experiments were plated for overnight growth from log phase growth, unless otherwise indicated. Immunofluorescence images were analyzed using ImageJ (NIH, Wayne Rasband, http://rsb.info.nih.gov/ij/).

### 4.2 Constructs and transient transfection

pTLxG‐PGC1a was generated by subcloning PGC‐1a cDNA (Anderson et al., 2008) into the pTLcG vector (Ko et al., 2011), and the interrupting GFP cassette was removed by in vitro Cre‐mediated recombination. pcDNA3.1‐PGC1a transfections were collected 24 hr after transfection. pTLxG‐PGC‐1a‐transfected cells were treated with doxycycline (Sigma‐Aldrich, D9891) at 0.1 μg/mL 24 hr after transfection and collected simultaneously after 6‐ or 24‐hr treatment.

### 4.3 Transcriptomics

Each RNA library was generated following Illumina TruSeq RNA Sample Preparation Guide and the Illumina TruSeq RNA Sample Preparation, with quality and quantity assessed using an Agilent DNA1000 series chip assay and Invitrogen Qubit HS Kit (Invitrogen), respectively. Sequencing reads were trimmed to remove sequencing adaptors and low‐quality bases (Jiang, Lei, Ding, & Zhu, 2014), aligned to mm10 reference genome (Ensembl release 85; Yates et al., 2016) using the STAR aligner (Dobin et al., 2013) and alignments used as input to RSEM for quantification (Li & Dewey, 2011), with differential gene expression analysis via EdgeR (Robinson, McCarthy, & Smyth, 2010) generalized linear model (GLM) method. Differential exon usage was detected via DEXSeq (Anders, Reyes, & Huber, 2012), filtered to only include exons with at least 10 counts on average in at least one group. KEGG pathway analysis was conducted via WebGestalt (Wang, Duncan, Shi, & Zhang, 2013) with significance determined by BH‐adjusted *p* < 0.0001. Redundant, nested pathways were removed by curation.

### 4.4 Bioassays: multiphoton laser scanning microscopy

Conducted as previously described (Pugh et al., 2013). *Lipid extraction and gas chromatography:* Conducted as previously described (Polewski et al., 2015). *Bioassays:* Fatty Acid Oxidation: Fatty acid oxidation rates were measured as previously described (Huynh, Green, Koves, & Hirschey, 2014). JC‐1, NAD and NADP, Seahorse, and Oxo‐Plates Respiration assays: Conducted according to the manufacturer's instructions. Oxo‐Plate assay was conducted in open air; therefore, values approach an equilibrium of oxygen saturation. *G‐Actin/F‐actin assay:* Detected using a commercially available kit (Cytoskeleton, Inc. #BK037). *Citrate Synthase:* Citrate synthase activity was measured using a commercially available colorimetric kit (Sigma‐Aldrich, CS0720). *Flow Cytometry:* Conducted using standard protocols (Darzynkiewicz & Juan, 2001). Specific details for each method are described in Supporting Information.

### 4.5 HMDP dataset

Genome‐wide gene expression data were obtained as described (Bennett et al., 2010; Parks et al., 2013, 2015). Gene–gene correlations for *Ppargc1a* were calculated using biweight mid‐correlation (Parks et al., 2013), where genes that correlated with *p* < 0.05 for at least one probe were considered significant. Overlap of significantly correlated genes between tissues was determined using R statistical software.

### 4.6 ENCODE algorithm

The ENCODE database was queried for factors predicted to bind PGC‐1 responsive genes using mean CHIP‐seq signal for each gene. Predictions were then validated using a bootstrapping algorithm and adjusted for multiple analysis using the Benjamini–Hochberg correction. Factors reaching *p* < 0.05 were considered significant. Protein–protein interaction between factors was visualized using STRING (Szklarczyk et al., 2015).

### 4.7 Statistics

All Student's *t* tests were two‐tailed. Outliers were identified by Grubb's test using a threshold of *p* < 0.05. One‐way ANOVA was conducted assuming Gaussian distribution and corrected for multiple comparisons using Tukey's test.

### 4.8 Code availability

All code used is available from the corresponding author upon reasonable request.

### 4.9 Data accessibility

All microarray data from this study are deposited in the NCBI GEO database (http://www.ncbi.nlm.nih.gov/geo/) under the accession number GSE42890, GSE16780, and GSE64908. All other datasets are available from the corresponding author upon reasonable request.

## CONFLICT OF INTEREST

JMD is a consultant for BioTechne and FORGE Bioscience and is cofounder of Galilei BioSciences. The remaining authors declare no conflict of interest.

## AUTHOR CONTRIBUTIONS

KNM and RMA conceptualized the study; KNM and AR contributed to methodology; KNM, JPC, PRH, MSB, SAM, SAH, NBJ, and TWR investigated the study; DMP, KWE, BWP, AR, JMD, and JMN provided resources; KNM and RMA wrote the manuscript; RMA acquired funding; and RMA supervised the study.
