# Supplementary material for: PGC‐1a integrates a metabolism and growth network linked to caloric restriction
Source: Aging Cell. 2019 Jul 3;18(5):e12999. doi: 10.1111/acel.12999 (PMC6718593; doi:10.1111/acel.12999)
Supplement: Supplementary file 10 [file ACEL-18-e12999-s010.docx]

**Supplementary Table 4:** Related to Figure 1 and Supplementary Figure 1.

|  | **Average abundance** | |  |  |
| --- | --- | --- | --- | --- |
| **Peptide** | **Vector** | **PGC-OE** | **log_2_FC (OE/V)** | **p-value** |
| H3: K18ac K23ac | 1.0779375 | 1.204321271 | 0.159946773 | 0.019568322 |
| H3: K18ac/K23ac | 13.36372653 | 14.83840423 | 0.151013582 | 0.027117846 |
| H3: K18me K23un | 0.166120138 | 0.18147106 | 0.127512521 | 0.117325534 |
| H3: K18un K23me1 | 0.061536217 | 0.074005396 | 0.266194707 | 0.015931905 |
| H3: K18un K23un | 85.33067961 | 83.70179805 | -0.027805924 | 0.023154783 |
| H3: K27me1 K36me1 | 2.073427998 | 2.818692781 | 0.443008293 | 0.002276929 |
| H3: K27me1 K36me2 | 7.536485959 | 10.15986208 | 0.430916918 | 1.2626E-05 |
| H3: K27me1 K36me3 | 2.957916781 | 3.570729049 | 0.271637201 | 0.009282632 |
| H3: K27me1 K36un | 9.712911792 | 7.256007384 | -0.420727936 | 1.02356E-05 |
| H3: K27me2 K36me1 | 6.45179362 | 10.63164619 | 0.720592803 | 1.59637E-05 |
| H3: K27me2 K36me2 | 1.536707711 | 2.998470754 | 0.964384117 | 4.7921E-06 |
| H3: K27me2 K36un | 35.25808658 | 32.32608602 | -0.125255344 | 0.000368337 |
| H3: K27me3 K36me1 | 1.914550106 | 2.580339939 | 0.430555724 | 4.70382E-05 |
| H3: K27me3 K36me2 | 0.273159151 | 0.587411985 | 1.104630948 | 8.00917E-05 |
| H3: K27me3 K36un | 8.979075629 | 7.658794872 | -0.229449532 | 0.000372203 |
| H3: K27un K36me1 | 2.779425252 | 2.911602784 | 0.067026966 | 0.385159748 |
| H3: K27un K36me2 | 7.331742589 | 6.970452133 | -0.072903897 | 0.318972765 |
| H3: K27un K36un | 13.19471684 | 9.529904029 | -0.4694268 | 0.000264565 |
| H3: K4me1 | 5.997675708 | 7.051929197 | 0.233614471 | 0.141041527 |
| H3: K4me2 | 0.391485467 | 0.364390074 | -0.10347509 | 0.694212501 |
| H3: K4me3 | 0.232898368 | 0.20006267 | -0.219248527 | 0.408735063 |
| H3: K4un | 93.37794046 | 92.38361806 | -0.015444721 | 0.114824252 |
| H3: K79me1 | 3.059361122 | 2.737940136 | -0.160139507 | 0.507520108 |
| H3: K79me2 | 12.32769603 | 10.62390351 | -0.214589246 | 0.595970162 |
| H3: K79un | 84.61294285 | 86.63815636 | 0.03412418 | 0.483899291 |
| H3: K9ac K14ac | 0.143040891 | 0.225537076 | 0.656936993 | 0.000573784 |
| H3: K9ac/K14ac | 4.418459069 | 5.286223229 | 0.258694029 | 0.024584496 |
| H3: K9me1 K14ac | 2.882237116 | 3.773007697 | 0.388526015 | 0.009308345 |
| H3: K9me1 K14un | 12.44913952 | 9.837944134 | -0.33961726 | 0.029226988 |
| H3: K9me2 K14ac | 6.828912062 | 10.86358234 | 0.669772258 | 0.000300482 |
| H3: K9me2 K14un | 31.75200534 | 33.59087111 | 0.081221499 | 0.008621349 |
| H3: K9me3 K14ac | 2.390771379 | 3.380963726 | 0.499958361 | 0.015484114 |
| H3: K9me3 K14un | 25.78151493 | 21.43737365 | -0.266208871 | 0.001169099 |
| H3: K9un K14un | 13.35391969 | 11.60449704 | -0.202579274 | 0.088620551 |
| H4: 1ac | 32.20187234 | 30.79592582 | -0.064405074 | 0.503836636 |
| H4: 2ac | 5.29708671 | 7.171894193 | 0.43715508 | 0.0012348 |
| H4: 3ac | 1.004611359 | 1.438494898 | 0.517922612 | 0.001976263 |
| H4: 4ac | 0.21012462 | 0.294468728 | 0.48686922 | 0.023016386 |
| H4: unmodified | 61.28630497 | 60.29921636 | -0.023425472 | 0.582361009 |
