# Supplementary material for: PGC‐1a integrates a metabolism and growth network linked to caloric restriction
Source: Aging Cell. 2019 Jul 3;18(5):e12999. doi: 10.1111/acel.12999 (PMC6718593; doi:10.1111/acel.12999)
Supplement: Supplementary file 11 [file ACEL-18-e12999-s011.docx]

**Supplementary Table 5:** Related to Figure 3. PGC-OE phospholipid percent composition ±SD.

|  | Phosphatidylcholines | | | Phosphatidylethanolamines | | |
| --- | --- | --- | --- | --- | --- | --- |
|  | Vector | PGC-OE | p-value | Vector | PGC-OE | p-value |
| 14:0 | 0.34±0.25 | 0.41±0.36 |  |  |  |  |
| 16:0 | 32.30±1.40 | 37.45±1.46* | 0.01 | 7.70±0.57 | 7.44±0.72 |  |
| 16:1n10 | 0.73±0.05 | 1.16±0.08* | 0.003 | 0.00±0.00 | 0.04±0.04 |  |
| 16:1n7 | 3.45±0.27 | 3.31±0.16 |  | 0.86±0.01 | 0.82±0.06 |  |
| 18:0 | 17.33±1.53 | 15.71±1.28 |  | 41.04±0.57 | 42.39±0.32* | 0.03 |
| 18:1n9 | 34.06±0.64 | 31.90±0.78* | 0.02 | 20.49±0.27 | 20.06±0.17 |  |
| 18:1n7 | 2.02±0.06 | 2.09±0.05 |  | 1.67±0.05 | 1.71±0.02 |  |
| 18:2n6 | 7.42±0.13 | 4.96±0.29* | 0.001 | 4.88±0.19 | 2.80±0.06* | 0.001 |
| 18:3n6 | 0.48±0.01 | 0.62±0.01* | <0.001 | 0.50±0.06 | 0.61±0.05 | 0.054 |
| 18:3n3 | 0.74±0.04 | 0.00±0.00* | 0.001 | 0.86±0.04 | 0.45±0.03* | <0.001 |
| 20:0 |  |  |  | 0.10±0.07 | 0.08±0.05 |  |
| 20:1n9 |  |  |  | 0.29±0.04 | 0.35±0.07 |  |
| 20:3n6 |  |  |  | 1.13±0.02 | 1.12±0.02 |  |
| 20:4n6 |  |  |  | 7.24±0.22 | 8.07±0.10* | 0.01 |
| 20:5n3 | 1.12±0.46 | 2.39±0.48* | 0.03 | 9.61±0.34 | 9.99±0.02 |  |
| 22:0 |  |  |  | 0.02±0.02 | 0.00±0.00 |  |
| 22:6n3 |  |  |  | 3.62±0.16 | 4.05±0.04* | 0.03 |

*Significance at P<0.05 PGC-OE versus Vector.
