# Supplementary material for: PGC‐1a integrates a metabolism and growth network linked to caloric restriction
Source: Aging Cell. 2019 Jul 3;18(5):e12999. doi: 10.1111/acel.12999 (PMC6718593; doi:10.1111/acel.12999)
Supplement: Supplementary file 12 [file ACEL-18-e12999-s012.docx]

**SUPPORTING INFORMATION**

**SUPPLEMENTARY EXPERIMENTAL PROCEDURES**

*Animals and diets:* This study was approved by the Institutional Animal Care and Use Committee at the University of Wisconsin, Madison. Four-week-old male B6C3F1 hybrid mice were purchased from Jackson Laboratories. Mice were individually caged and fed the AIN-93M semi-purified diet. Mice were randomized into two groups at 2 months of age. Control mice (CN) were fed 87 kcal/week which is 95% of ad libitum. Calorie restricted animals (CR) received a 25% reduction from controls. Mice were individually housed to ensure consumption of all food and that precise caloric intake could be known. Mice were euthanized by cervical dislocation. Tissues were isolated, fixed in formalin and paraffin embedded or flash frozen in liquid nitrogen and stored at -80°C until further processing.

*Insulin and adiponectin analysis:* Commercially available ELISA kits were used to detect circulating levels of insulin (80-INSMS-E01; Alpco) and adiponectin (DY1119; R&D Systems). High molecular weight adiponectin isoforms were distinguished from total adiponectin levels by proteinase K digest.

*Cell Culture:* 3T3-L1 cells were obtained from ATCC and maintained in DMEM (Gibco) with 10% bovine serum (Gibco) and 1% penicillin/streptomycin (Sigma Aldrich). pcDNA3.1-PGC1a cDNA (D. Kelly, WUSTL) was subcloned into lentiviral transfer vector pWPXL (Addgene), and 3T3-L1 cells stably overexpressing PGC-1a were generated by pWPXL-PGC-1a viral delivery. Clonal cell lines were isolated and assessed by PGC-1a expression. The PGC-OE clone used had no overall change in adipogenic gene expression (Fig.S1J). Cells used in experiments were plated for overnight growth from log phase growth, unless otherwise indicated. Cells in reserve were maintained every two days, and doubling time was calculated with each passage. NIH-3T3 cells were maintained under the same conditions and NIH-3T3 PGC-OE cells were generated by transfection of pcDNA3.1-PGC-1a construct (gift of D. Kelly, WUSTL) or empty vector.

*Western Blot and Immunofluorescence:* Protein was extracted from cultured cells using modified RIPA buffer, and western blotting was done using standard techniques (Anderson et al., 2008) and detected by enhanced chemiluminescence using an ImageQuant LAS4000 (General Electric). Immunofluorescence was carried out using standard techniques. Briefly, cells were grown overnight on glass coverslips and fixed in 3.7% formaldehyde/PBS for 10 minutes. Cells were permeablized with 0.3% Triton X-100 in PBS, blocked with 1% BSA solution, and incubated overnight in primary antibody. Proteins were visualized using fluorescently tagged secondary antibodies (Vector Laboratories, FL-1000 and FL-2000) and mounted onto glass slides using Vectashield (Vector Labs) hard-mount mounting solution. Neutral lipids were stained with BODIPY 493/503 dye (Thermo Fisher, D3922). Filamentous actin was visualized using rhodamine-labeled phalloidin (Cytoskeleton Inc., PDHR1). Images were captured on a Leica DM4000B microscope using a Retiga 4000R digital camera (QImaging systems). Antibodies used included PGC-1a (Santa Cruz Biotechnology, 13067); Thermo-Fisher: SIRT1 (A13973); Sigma-Aldrich: Tubulin (T9026), Actin (A1978); Abcam: OXPHOS cocktail (ab110413), VDAC (ab14734), TOMM20 (ab56783); Cell Signalling: pAMPK (#2535), AMPK (#2532), RAPTOR (#2280), RICTOR (#9476), pAKT S473 (#4060), pAKT T308 (#4056), AKT pan (#4685), pGKS-3b (#9336), GSK-3b (#9315), pERK (#4376), ERK (#4695), pIRS1 S307 (#2381), IRS1 (#2382), pS6 (#2211), S6 (#2217), and COX4 (#4850).

*Quantitative image analysis:* Immunofluorescence images were analyzed using Image J (NIH, Wayne Rasband, http://rsb.info.nih.gov/ij/). Mitochondrial morphology and lipid droplet size analyses were conducted on images after background subtraction and thresholding on a per-cell basis. Cell size was estimated using tubulin immunostaining, and similar results were obtained by F-actin fluorescent staining (not shown). Tubulin cytoskeletal analysis was done on images after background subtraction and thresholding using the Skeleton Analysis plugin with the “shortest branch” parameter.

*Multiphoton laser scanning Microscopy:* Immediately prior to multiphoton imaging, cells grown overnight on glass coverslips were fixed for 60 minutes with formalin and mounted onto glass slides using Vectashield (Vector Labs) hard-mount mounting solution. The instrument response function of the optical system was calibrated before each imaging session. A Nikon 100X oil immersion objective (Melville, NY, USA) was used for 3T3-L1 PGC-OE cells and a 20X air objective was used for NIH-3T3 PGC-OE cells. Data were collected using an excitation wavelength of 780 nm, and emission was filtered using a 457 ± 50 nm bandpass filter (Semrock, Rochester, NY), the spectral peak for NADH/NADPH. FLIM images were collected at 256x256 resolution with 120 second collection using SPC-830 Photon Counting Electronics (Becker & Hickl GbmH, Berlin, Germany) and Hamamatsu H7422P-40 GaAsP photomultiplier tube (Hamamatsu Photonics, Bridgewater, NJ). Urea crystals were used to determine the Instrumentation Response Function (IRF) with a 390/10 bandpass emission filter (Semrock, Rochester. NY). Autofluorescence intensity and fluorescence lifetime data were analyzed in SPCImage (Becker & Hickl, v.3.9.7, Berlin, Germany) where a Levenberg–Marquardt routine for nonlinear fitting was used to fit the fluorescence decay curve collected for each pixel in the 256 × 256 frame to a model multi‐exponential decay function. Data were assessed by the minimized chi‐square value generated during the fit so that analysis was unbiased. To eliminate background fluorescence a threshold for analysis was applied based on photon counts. Additionally, pixels were assigned a bin of 2 for optimal fitting of the data. For NAD(P)H autofluorescent intensity, data were analyzed in ImageJ (NIH, Wayne Rasband, http://rsb.info.nih.gov/ij/) and regions were defined by cellular compartment. For fluorescence lifetime, regions of interest were defined by the same criteria using the inclusion tool in SPC image.

*qRT-PCR:* Cells were lysed in Trizol (Thermo Fisher, 15596018), and RNA was isolated using a commercially available kit (Zymo Research, R2072). cDNA was synthesized using a commercially available high capacity kit (Thermo Fisher, 4368813). Quantitative real time PCR was performed on an Applied Biosystems StepOnePlus or Quant Studio 3 using iTaq SYBR green master mix reagent (BioRad, 1725150). Primers for PGC-1a isoforms have been described previously(Ruas et al., 2012). PGC-1a transcript detection otherwise used primers to detect both a1 and a4 isoforms, F: AGCCGTGACCACTGACAACGAG, R: GCTGCATGGTTCTGAGTGCTAAG. Expression was normalized using the ΔΔCt method to 18S with primers F: GTAACCCGTTGAACCCCATT, R: CCATCCAATCGGTAGTAGCG.

*Lipid extraction and gas chromatography:* Lipids were extracted from 15cm plates of cells following a modified Folch method (Folch, Lees, & Sloane Stanley, 1957). Pentadecanoic acid was added as an internal control of transmethylation efficiency. Neutral lipid species and PL lipid species were separated on silica gel-60 TLC plates (EMD Millipore) using a heptane/isopropyl ether/acetic acid (60/40/3) solvent system. PL bands were scraped from plates, lipids were extracted and transmethylated using boron trifluoride in 14% methanol (Sigma). Fatty acid methyl esters were resuspended in hexane and analyzed by gas liquid chromatography. Chromatograms were analyzed using HP ChemStation software. Results were calculated to express fatty acid as percent composition.

*Fatty Acid Oxidation:* Fatty acid oxidation rates were measured as previously described (Huynh, Green, Koves, & Hirschey, 2014). Briefly, cells grown on 6 well plates overnight then subsequently incubated for 3 hours with a reaction mixture containing 14C-labelled palmitate. Perchloric acid was added to stop the reaction, and unoxidized palmitate was precipitated out of solution by centrifugation. CO_2_ generated from acetyl-CoA oxidation was captured by 1M NaOH into Whatman filter paper. 14C –containing acid soluble metabolites (ASMs) and trapped CO_2_ were measured in a liquid scintillation counter. The total fatty acid oxidation values reported reflect the sum of the CO_2_ and the acid soluble metabolite fractions.

*JC-1:* PGC-OE and control cells were grown overnight on a clear-bottom black 96-well plate, then incubated in JC-1 dye (Thermo Fisher, T-3168) at 1.5 μM for 30 minutes. After washing twice with PBS, fluorescence emission was measured through the bottom of the plate at 590 nm and 530 nm with excitation at 535 nm and 485 nm.

*NAD and NADP assays:* Levels of the coenzymes were quantified using colorimetric assay kits for NAD+/NADH (Biovision, K337) and NADP+/NADPH (Biovision, K347).

*Seahorse assay:* Cells were plated overnight at 7500 cells/well in a 0.1% gelatin-coated Seahorse assay plate. Mitochondrial fuel usage was determined using a Seahorse XF mito fuel flex test kit (Agilent Technologies, 103260-100) on a Seahorse XFe96 analyzer according to the manufacturer’s instructions.

*Respiration Assay:* Oxygen consumption was measured using Oxo-Plates (Presens, #OP96U) according to manufacturer’s instruction with slight modification. Respiration buffer (Barrientos, 2002) was used instead of water for preparing the calibrations controls and cell samples. Cells were grown overnight, resuspended in respiration buffer, and plated at 5x10^5^ cells per well on to the Oxo-Plate along with calibration controls (ambient k100) and (anoxic k0). Fluorescence was detected over time (Lm1: Ex.540 Em. 650; Lm2: Ex.540 Em.590) using a fluorescence plate reader (Molecular Devices SpectraMax M3). pO2 (%air saturation) was calculated using the following equation: pO2 = 100 x (k0 / IR-1) / (k0 / k100 - 1) where IR is unknown. The assay was conducted in open air; therefore, values approach an equilibrium of oxygen saturation.

*G-Actin/F-actin assay:* Relative amounts of globular and filamentous actin were detected using a commercially available kit (Cytoskeleton, Inc. #BK037).

*Citrate Synthase:* Citrate synthase activity was measured using a commercially available colorimetric kit (Sigma Aldrich, CS0720).

*Flow Cytometry:* Cells were plated for two days of growth, then replated for overnight growth, fixed in 70% ethanol, stained with propidium iodide using standard protocols (Darzynkiewicz & Juan, 2001). Samples were run on a FACSCalibur benchtop system (BD Biosciences), manually gating to detect the cell population of interest (10,000 cells). Data were analyzed using ModFit LT software v4.1 (Verity Software House).

*Constructs and transient transfection:* Cells in 35mm plates at 80% confluence were transfected with 2 μg expression plasmid or empty vector control DNA using 10 μL of Lipofectamine 2000 per well according to the manufacturer’s protocol (Thermo Fisher, 12566014). pcDNA3.1-PGC1a was a gift of D. Kelly, WUSTL. pTLxG-PGC1a was generated by subcloning PGC-1a cDNA into the pTLcG vector (Ko et al., 2011), and the interrupting GFP cassette was removed by *in vitro* Cre-mediated recombination. pcDNA3.1-PGC1a transfections were collected 24 hours after transfection. pTLxG-PGC-1a transfected cells were treated in reverse with doxycycline (Sigma Aldrich, D9891) at 0.1 μg/mL 24 hours after transfection and collected 6 and 24 hours later.

*Transcriptomics:* PGC-OE cells were homogenized in TRIzol. RNA extraction proceeded using a Direct-zol RNA kit (Zymo Research) according to the manufacturer’s instructions. Each RNA library was generated following Illumina TruSeq RNA Sample Preparation Guide and the Illumina TruSeq RNA Sample Preparation Kit (Illumina Inc.). Quality and quantity of finished libraries are assessed using an Agilent DNA1000 series chip assay (Agilent Technologies) and Invitrogen Qubit HS Kit (Invitrogen), respectively. Sequencing reads were trimmed to remove sequencing adaptors and low quality bases using skewer (Jiang, Lei, Ding, & Zhu, 2014) trimming program with a minimum adapter overlap of 15, minimum base quality of Q20. Reads shorter than 25 nucleotides after trimming were removed from downstream analysis. The trimmed sequences were aligned to the annotated (Ensembl release 85 (Yates et al., 2016)) mm10 reference genome using the STAR aligner (Dobin et al., 2013). Transcriptome alignments from STAR were used as input to RSEM for quantification(B. Li & Dewey, 2011). The expected read counts from RSEM were used for differential gene expression analysis using EdgeR(Robinson, McCarthy, & Smyth, 2010). Lowly expressed genes were filtered by requiring at least 3 samples to have more than 1 count per million reads sequenced (cpm>1). EdgeR’s default normalization was used to calculate the normalization factors which correct for the different sequencing depth of each library. We used EdgeR’s generalized linear model (GLM) method to infer differential expression between conditions. Detection of differential exon usage was done by using the DEXSeq (Anders, Reyes, & Huber, 2012) package to analyze the STAR genome alignments. DEXSeq output was filtered to only include exons with at least 10 counts on average in at least one group. KEGG pathway analysis was conducted using WebGestalt (Wang, Duncan, Shi, & Zhang, 2013) with significance determined by BH-adjusted p<0.0001. Redundant, nested pathways were removed by curation (Supplementary Table 3). The Hallmark Adipogenesis gene set was obtained from the Molecular Signatures Database (MSigDB: http://software.broadinstitute.org/gsea/msigdb), and the PPARG Target Gene set was obtained from the PPARgene database (http://www.ppargene.org/) (Fig.S1).

*HMDP dataset:* Genome-wide gene expression data was obtained from flash-frozen liver, epididymal adipose and skeletal muscle samples as described (Bennett et al., 2010; Parks et al., 2013; Parks et al., 2015). Gene-gene correlations for Ppargc1a were calculated using biweight midcorrelation, which is robust to outliers (Parks et al., 2013). Genes that correlated with p<0.05 for at least one probe were considered significant. Data from skeletal muscle was only available from mice fed a high fat diet; however, high fat diet feeding had little effect on gene correlation with PGC-1a (data not shown). Overlap of significantly correlated genes between tissues was determined using R statistical software.

*ENCODE algorithm:* The algorithm queries the ENCODE database with an input gene set and outputs ENCODE factors predicted to bind the input genes using mean CHIP-seq signal for each gene. Predictions were then validated using a bootstrapping algorithm. P values were corrected for multiple analysis using the Benjamini-Hochberg correction. Factors reaching p < 0.05 were considered significant. Protein-protein interaction between factors was visualized using STRING (Szklarczyk et al., 2015) with medium (>0.400) or high (>0.700) confidence thresholds (as indicated) based on textmining, experiments, databases, neighborhood, gene fusion, co-occurrence, and co-expression sources.

*Statistics:* All Student’s t-tests were two-tailed. Outliers were identified by Grubb’s test using a threshold of p < 0.05. One-way ANOVA was conducted assuming Gaussian distribution and corrected for multiple comparisons using Tukey’s test.

*Code availability:* All code used is available from the corresponding author upon reasonable request.

**SUPPLEMENTARY FIGURE LEGENDS**

Supplementary Figure 1: Related to Figure 1. (A) Western blot detection of PGC-1a and quantitation, n=3. (B) qRT-PCR detection of PGC-1a isoforms in RNA samples used for RNA-seq, n=4. (C) Principal components analysis. (D) Top upregulated and downregulated genes in the RNA-seq dataset. (E) Expression of KEGG fatty acid-related pathway genes by RNA-seq, n=4. (F) Quantitation of PGC-1a mRNA levels by qRT-PCR, n=4, and expression of PGC-1a target genes by RNA-seq. (G) Doxycyline-inducible (0.1 μg/mL) expression of PGC-1a and response of target genes by qRT-PCR, n=6. (H) qRT-PCR detection of PGC-1a expression and target genes in response to transient transfection of PGC-1a cDNA, n=6. (I) Heatmap of histone modifications in PGC-OE versus Vector cells, n=6. (J) Heatmaps of adipogenesis and PPARG target genes by RNA-seq, n=4 (*see also* Supplemental Table 1). Data shown as means ± SEM; asterisk (*) indicates p<0.05 by two-tailed Student’s t-test or differential expression (F).

Supplementary Figure 2: Related to Figure 2. (A) Western blot detection of mitochondrial electron transport chain subunits, n=3. (B) Expression of KEGG OXPHOS pathway genes by RNA-seq, n=4. (C) Oxygen consumption rate changes in response to BPTES, UK5099, and etomoxir (Eto) and calculations for dependency and capacity, n=6. Data shown as means ± SEM; asterisk (*) indicates differential expression.

Supplementary Figure 3: Related to Figure 4. Means and distributions of (A) τ_1_, fluorescence lifetime of free NAD(P)H and (B) τ_2_, fluorescence lifetime of bound NAD(P)H, n=9 Vector and 10 PGC-OE. Ratios of nuclear to cytosolic (C) NAD(P)H fluorescence intensity, n=9 Vector and 11 PGC-OE and (D) mean fluorescence lifetime (τ_m_), n=9. Data shown as means ± SD; asterisk (*) indicates p<0.05 by two-tailed Student’s t-test.

Supplementary Figure 4: Related to Figure 5. (A) Representative flow cytometry histogram and scatter for the data shown in Fig.5b. (B) Pathway schematic of genes with differential exon expression (orange outlines) involved in growth signaling and cytoskeletal regulation. (C) Exon expression of Mtor and sashimi plot. (D) Representative western blot detection of filamentous (F) and globular (G) actin and quantitation, n=3. (E) Representative western blots of growth signaling mediators, n=3. Data shown as means ± SEM.

Supplementary Figure 5: Related to Figure 6. (A) Overlap of transcripts correlated (green), positively correlated (red) or negatively correlated (blue) with PGC-1a expression in three different tissues (biweight midcorrelation p<0.05).

Supplementary Figure 6: PGC-OE clones in NIH-3T3 cells. (A) Western blot of detection of PGC-1a in whole-cell lysate and (B) quantitation of clone 3 used in following experiments, n=4 experiments. (C) JC-1 measurement of mitochondrial membrane potential, n=16. (D) Oxo-plate respiration assay, n=7. (E) NAD biochemical assay, n=3. (F-H) Quantitation of means (left) and distributions (right) of NAD(P)H fluorescence lifetime of whole-cell measurements, n=4. Data shown as means ± SEM (A-E) or ±SD; asterisk (*) indicates p<0.05 by two-tailed Student’s t-test.
